# 

**Published:** 1900-07

**Authors:** 


### INDEX TO VOL. XVI.

Abstracts and Selections..........22-28, 73-76, 123-124, 171-172, 201-208, 388-392, 492, 533-534
Against Substitution.......... 537
American Congress of Tuberculosis..........442, 491
Anesthesia in Labor.......... 193
Annual Address of the President of the Texas Academy of Science. 281
Annual Meeting of the Association of Medical Officers of the Confederacy.......... 386
Another District Medical Society Organized.......... 170
An Entering Wedge,—Perhaps.......... 128
Austin District Medical Association.......... 255
A Labor Saver.......... 214
A Merry Christmas Doctor.......... 261
A Notable Address on State Medicine.......... 125
A Report of Four Surgical Cases.......... 329
A Serious Farce.......... 34
A State Sanatorium for Consumptives.......... 261

Books and Magazines..........39-42, 86-93, 133-137, 180-181, 218-223, 271-274, 318-322, 369-370, 404-408, 498-508
Brazos Valley Medical Association..........22, 198, 252, 442, 490, 523
Breitenbach & Co.'s Fire.......... 214

Catarrhal Fever.......... 383
Central Texas Medical Association..........19, 303
Chlorotone as a Sedative in Gastric and Cystic Irritability.......... 384
Clinical Notes..........46-48
Correspondence..........14-19, 164, 169, 428

Dr. A. N. Denton.......... 393
Dr. Edwin Pinckney Becton.......... 307
Dr. Preston B. Scott.......... 82
Dr. Russ.......... 443
Dr. Welch's Article on Smallpox.......... 355

East Texas Medico-Chirurgical Society..........199, 303, 491, 530
Editorial Notes..........173-174, 309-310
El Paso County Medical Society.......... 200

From a Subscriber.......... 169

General Medicine.......... 243

Health and Rest.......... 130

Indigestion .......... 356
Injection Treatment of Hernia .......... 71
In the Matter of a State Sanatorium for Indigent Consumptives... 311
I. N. Love, M. D .......... 29

Medical Association of Texas, New Mexico, Arizona and Mexico... 303
Medical Legislation .......... 359, 397
Mississippi Valley Medical Association .......... 123, 303
Modern Medical Training .......... 237

News and Miscellany .......... 35-36, 83-86, 131-132, 175-179, 215-218, 268-271, 314-318, 367-369, 401-404, 444-445, 496-498, 537-541
Neurasthenia .......... 114
New York Academy of Medicine .......... 256
News Items Concerning Third Pan-American Medical Congress..... 304
North Texas Medical Association .......... 256

Panhandle Medical Association .......... 123
Pan-American Medical Congress .......... 254, 263
Pelvic Abscess .......... 513
Pernicious Vomiting of Pregnancy and its Treatment with Electricity .......... 60
Placenta Prævia .......... 426
Post Partum Hemorrhage .......... 211
Public Standing of the Physician .......... 469
Publisher's Notes .......... 42-46, 94-95, 137-142, 181-192, 223-239, 274-280, 323-328, 370-372, 408-416, 446-460, 508-512, 542-544

Reform in Asylum Laws and Management .......... 32
Report of Chairman of Section on Obstetrics and Gynecology .......... 246
Remarks on the Present Mild Type of Smallpox; the Symptoms and Diagnosis .......... 341

San Antonio New Hospital .......... 535
Scarlatina or Something Similar .......... 108
Should the Use of Antitoxin in Tuberculosis be Condemned from a Purely Scientific Point of View .......... 56
Some Surgical Cases .......... 97
Some Requisites for Successful Work in Obstetrics and Diseases of Children .......... 156
Southern Surgical and Gynecological Association .......... 170
South Texas District Medical Association .......... 256
South Texas Medical Association .......... 530, 532
Smallpox at Terrell Asylum .......... 264
Section on Obstetrics, Texas State Medical Association .......... 387
Some Remarks on Abdominal Surgery .......... 420
State Association of Health Officers—Meeting .......... 429

Tenth Semi-Annual Meeting of the South Texas Medical Association .......... 532
Texas State Medical Association .......... 386, 435, 461

The Physician as a Sanitarian........ 1
The Country Doctor........ 10
The Correct Thing........ 34
The Treatment of Phthisis Pulmonalis of Tubercle Bacilli........ 49
The Clinical Laboratory as an Adjunct to Medicine........ 64
The Baby's Second Summer........ 67
The "Newer Psychology"........ 77
The Old Man Must Go........ 102
The "Campaign of Education"........128, 209
The Mosquito as a Transmitter of Micro-Organisms........ 143
The Great Storm at Galveston........ 164
The Cause and Treatment of Puerperal Sepsis........ 196
The Western Texas Medical Association........ 200
The Preparation of the Lying-in Patient and the Management of Normal Labor........ 249
The Plague Situation at San Francisco........ 263
The Fine Old Irish Surgeon........ 267
The Man Who Knew It All........ 362
The Roberts-Hawley Goat Lymph Compound........ 373
The New Medical Practice Act........ 394
The Association of Texas Health Officers........ 444, 486
The State Medical Association........ 493
Transactions Texas State Medical Association for 1900........ 214
Treatment of the Insane........ 149
Tri-State Medical Society of Alabama, Georgia and Tennessee........ 160
Two Unique Cases of Renal Hemorrhage........ 417

Uric Acid Toxaemia........ 117

Ye Editor's Vacation........ 129
Yellow Fever in Mississippi........ 265

